# Efficient use of discarded vegetal residues as cost-effective feedstocks for microbial oil production

**DOI:** 10.1186/s13068-023-02268-5

**Published:** 2023-02-09

**Authors:** María Gallego-García, Antonio D. Moreno, Alberto González, María José Negro

**Affiliations:** 1 Advanced Biofuels and Bioproducts Unit, CIEMAT, Avda. Complutense 40, 28040 Madrid, Spain; 2 Universidad de Alcalá, Alcalá de Henares, 28805 Madrid Spain

**Keywords:** Single cell oil, Agri-food residues, Oleaginous yeast, Lipids

## Abstract

**Background:**

Horticultural intensive type systems dedicated in producing greenhouse vegetables are one of the primary industries generating organic waste. Towards the implementation of a zero-waste strategy, this work aims to use discarded vegetables (tomato, pepper and watermelon) as feedstock for producing microbial oil using the oleaginous yeast *Cryptococcus curvatus*.

**Results:**

The soluble fraction, resulting after crushing and centrifuging these residues, showed *C*/*N* ratios of about 15, with a total carbohydrate content (mainly glucose, fructose and sucrose) ranging from 30 g/L to 65 g/L. Using these liquid fractions as substrate under a pulse-feeding strategy with a concentrated glucose solution resulted in an intracellular total lipid accumulation of about 30% (*w*/*w*) of the total dry cell weight (DCW). To increase this intracellular lipid content, the initial *C*/*N* content was increased from 15 to 30 and 50. Under these conditions, the process performance of the pulse-feeding strategy increased by 20–36%, resulting in a total intracellular lipid concentration of 35–40% DCW (*w*/*w*).

**Conclusion:**

These results demonstrate the potential of discarded vegetables as a substrate for producing bio-based products such as microbial oil when proper cultivation strategies are available.

**Supplementary Information:**

The online version contains supplementary material available at 10.1186/s13068-023-02268-5.

## Background

Microbial oils are suggested as a suitable alternative to petroleum-based chemistry to reduce current environmental pollution. Microbial oils, also referred to as single cell oils (SCO), are produced by oleaginous microorganisms (bacteria, fungi, microalgae and yeast) capable of accumulating > 20% (*w*/*w*) lipids per dry cell weight (DCW) under particular cultivation conditions [1, 2]. The fatty acids (FA) profile of SCOs can vary depending on the microorganism and carbon source, making it highly suitable and valuable for diverse industrial applications, including the production of biofuels and the oleo-chemical sector [3].

Oleaginous yeasts, under conditions of nutrient limitation, can accumulate more than 70% of lipids to its biomass weight [4] and are characterized by its excellent accumulation capacity of FA in the form of triglycerides, which are valuable precursors for biodiesel production [5]. For these reasons, these microorganisms are of great interest in bioprocessing, and it must be characterized under industrial-like conditions to investigate its full potential.

Yeasts have certain advantages over other oil-producing organisms, such as plants, bacteria, microalgae and fungi. For instance, when compared to plants, yeasts have shorter growth times, fewer labor requirements, are not influenced by climate conditions, and do not occupy large land areas [2, 6, 7]. Yeasts are less influenced by climate changes when compared to microalgae, and it can utilize a wider range of carbon sources. Yeasts also exhibit greater tolerance to metal ions and have low oxygen demand when compared to fungi, and it is easy to harvest when compared to bacteria due to its larger size [8, 9]

Several oleaginous yeast species belonging to the genera *Cryptococcus*, *Lipomyces*, *Rhodosporidium* and *Yarrowia* can produce SCO and other relevant products (*e.g.*, carotenoids) depending on the culturing conditions. Temperature, C/N ratio, and pH are important parameters during SCO production, and these parameters must be optimized to maximize intracellular lipid accumulation [1, 10, 11]. In addition to the culturing conditions, low-cost substrates are highly important for cost-competitive microbial oil production [8, 12]. In recent years, different sources derived from food waste as carrot and apple pomace [13, 14], orange peels [15] or pumpkin peels wastes [16] have been used for yeast cultivation and lipids production. For example, Donzella et al. [16] report the utilization of pumpkin hydrolysate by enzymes without adding nutrients as media for lipid production by *Cutaneotrichosporon oleaginosum*, allowing producing of 24 g/L of biomass with 29% of lipids accumulation.

The horticultural intensive type systems dedicated in producing greenhouse vegetables represent one of the primary industries generating organic waste, especially in countries from South-Western Europe, such as Spain. These wastes include fruits that do not meet the quality standards required for sale and are typically removed during harvesting. In Europe, the losses of fruit and vegetable-derived from handling, storage and transport are around 5% of the total production. These residues cannot be stored for a long time due to its high moisture content. Today, these wastes are mainly used for animal feeding (*e.g.*, cattle and sheep) whenever possible. Nevertheless, in areas with no livestock farms, these residues are transferred to external waste management companies and/or authorized recycling plants for treatment, which implies an extra cost [17].

Discarded vegetables are rich in carbohydrates (50–80% w/w) and other nutrients (*e.g.*, nitrogen and vitamins). In this context, using these residues as feedstocks for yeast cultivation represents an attractive alternative with potential application in the energy sector. Carbohydrates from discarded vegetable products can be easily extracted by collecting the corresponding juice after a crushing procedure [18]. In this work, discarded pepper, tomato and watermelon were studied as raw material for accumulating intracellular lipids using the oleaginous yeast *Cryptococcus curvatus*. With the aim of maximizing lipid accumulation, these media were supplemented with a sugars-rich solution to increase the initial *C*/*N* ratios.

Furthermore, a pulse-feeding cultivation strategy was also evaluated for each substrate. All the different cultivation processes were evaluated regarding total lipid production, intracellular lipid content and overall conversion yield. In addition, the FA profile was investigated in order to assess the potential use of the resulting lipids to produce biodiesel. The results presented herein will contribute to optimizing the use of discarded vegetables as a substrate to produce SCO towards implementing a zero-waste Bioeconomy strategy.

## Results and discussion

### Discarded vegetable residues composition

The chemical composition of discarded vegetable residues (i.e., tomato, watermelon and pepper) ranged a total potential sugar content of 58–81% (*w*/*w*), thus, highlighting the potential of these residues to serve as a carbon source in different bioprocesses (Additional file 1: Table S1). The discarded vegetables were subjected to homogenization and liquid/solid separation to collect soluble carbohydrates. The resulting liquid fractions mainly contained glucose, fructose and sucrose as major sugars components (98% of the total sugars analyzed) (Table 1). Collected sugars in the liquid fraction accounted to 40.5% (*w*/*w*) (pepper), 39.4% (*w*/*w*) (tomato) and 63.3% (*w*/*w*) (watermelon), being discarded watermelon and discarded tomato the residues with the highest (68.8 g/L) and lowest (27.0 g/L) sugar concentrations, respectively (Table 1). In addition to sugars, nitrogen is another important component in bioprocessing cultivation media. The nitrogen concentration in discarded vegetable residues varied depending on the crop, ranging from 0.7 to 1.7 g/L (tomato < watermelon < pepper).

**Table 1:** Soluble fraction composition from discarded vegetables residues

Component (*g*/*L*)	Tomato	Watermelon	Pepper
Glucose	11.8 ± 0.9	14.5 ± 1.4	24.3 ± 0.8
Xylose	0.1 ± 0.0	0.1 ± 0.0	0.4 ± 0.0
Galactose	0.1 ± 0.0	0.1 ± 0.0	0.4 ± 0.1
Arabinose	–	–	–
Mannose	0.1 ± 0.0	0.1 ± 0.0	0.4 ± 0.2
Fructose	14.9 ± 1.3	29.3 ± 1.6	35.7 ± 1.5
Sucrose	–	24.3 ± 6.6	0.5 ± 0.2
Mannitol	–	0.3 ± 0.0	0.3 ± 0.1
Total nitrogen	0.7 ± 0.0	1.5 ± 0.02	1.7 ± 0.1

### Effect of C/N ratio on intracellular lipid accumulation

**Fig. 1:**
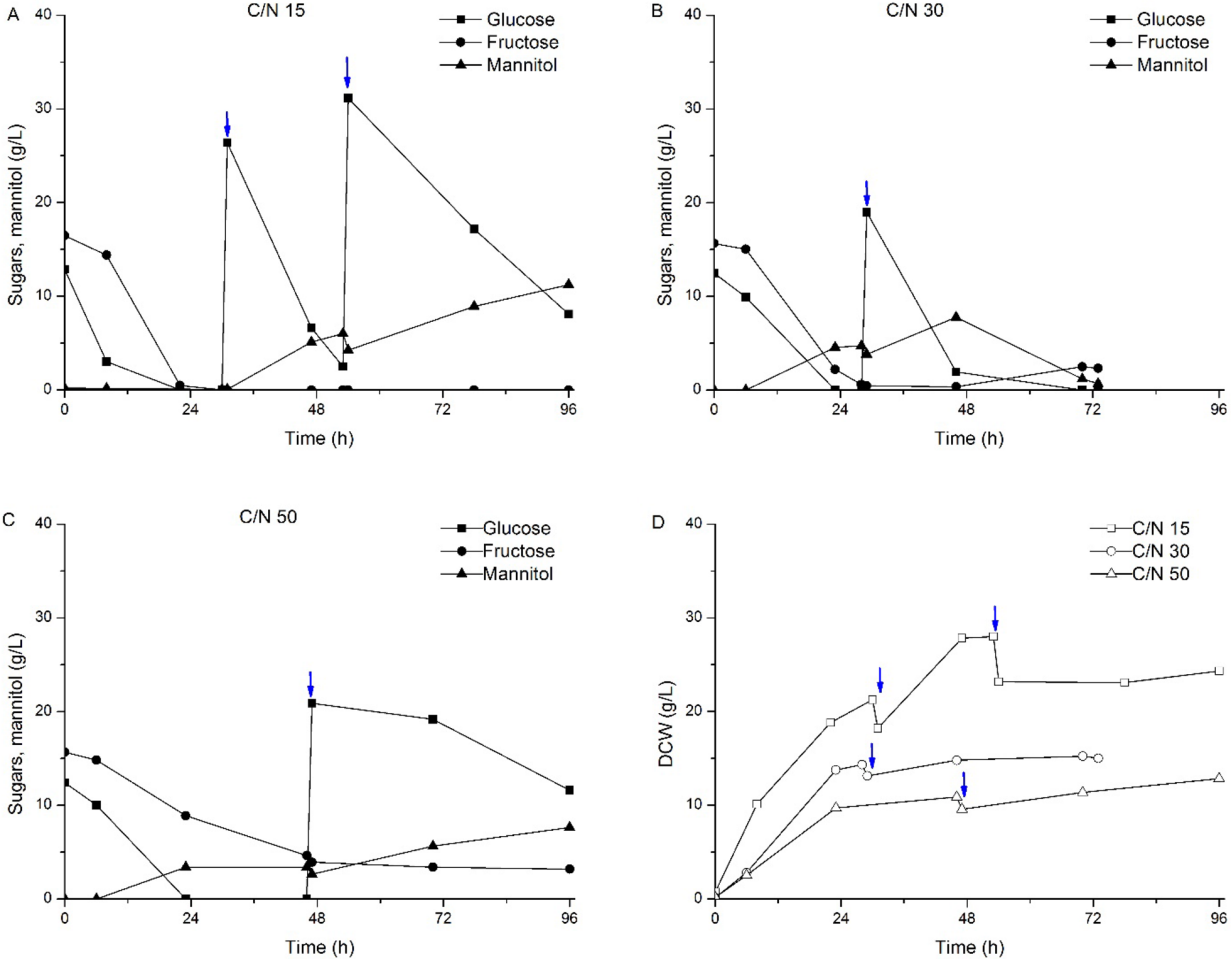
Time-course for sugars consumption and mannitol production from tomato-derived media for different *C*/*N* ratios and cell biomass production. ↓ Glucose pulse addition

After characterization, the collected liquid fractions were used to cultivate the oleaginous yeast *C. curvatus* for microbial oil production. Due to the impact of the C/N ratio on the intracellular lipid accumulation, the liquid fractions were mixed with a sugar-rich solution to increase the initial C/N ratio from 15 to 30 and 50 but maintaining the same sugar concentrations as in the raw material. Figure 1 depicts the sugar consumption and cell biomass production during the cultivation of *C. curvatus* in discarded tomato-derived media at different initial C/N rates. When using this media at an initial C/N ratio of 15, more than 76% of the initial glucose was consumed within the first 8 h, whereas 86% of the initial fructose was still present at this time. These sugar consumption rates decreased when increasing the C/N ratio of tomato-derived media to 30 and 50 (Fig. 1B, C), delaying complete glucose consumption up to 24 h. At this time point, fructose consumption was about 85% and 40% on media with initial C/N ratio of 30 and 50, respectively. Cell biomass concentration also varied when changing the initial C/N ratio (Fig. 1D). In this context, cell biomass concentration at C/N 15 (21.3 g/L at 30 h) almost doubled the cell concentration obtained at *C*/*N* 50 (10.8 g/L at 46 h) within shorter cultivation times, mainly due to the lower nitrogen content in the latter. This result is indicative of the nutrient-rich composition of the liquid fraction obtained from discarded tomato waste, so it supports cell growth without the need of supplementing the media with additives.

The total lipid content was analyzed after cultivating *C. curvatus* in tomato-derived media at different initial *C*/*N* ratios. In general, independently of this parameter, the lipid content was very low for an oleaginous microorganism [19]. Notwithstanding, the lipid content increased from 9.2% in cells collected from the *C*/*N* 15 media to 14.2% and 21.6% (*w*/*w*) in cells grown at *C*/*N* 30 and 50, respectively, while the lipid concentration remained approximately the same, from 2.6 g/L to 2.3 g/L and 2.6 g/L (Table 2). The higher lipid content in cells collected from media with an initial C/N ratio of 50 underlines the importance of increasing the initial C/N ratio to trigger lipid accumulation, as already reported in literature [20].

**Table 2:** Cell biomass yield, lipid production by *C. curvatus* cultured on Fed-batch pulse-feeding mode in discarded vegetable residues

Waste media	*C*/*N*		DCW yield (g/g)	Lipid production (g/L)	Lipid content (*w*/*w* %)	Lipid yield (*g*/*g* %)	Total Fame (%)
Tomato	15	Before 1st pulse	0.78 ± 0.02	2.6 ± 0.02	9.2 ± 0.1	0.090 ± 0.001	8.1 ± 0.2
		Final	0.45 ± 0.01	8.8 ± 0.04	29.4 ± 1.3	0.114 ± 0.000	24.3 ± 0.8
	30	Before 1st pulse	0.51 ± 0.03	2.3 ± 0.04	14.2 ± 0.2	0.082 ± 0.001	14.1 ± 0.1
		Final	0.36 ± 0.02	4.7 ± 0.1	29.2 ± 0.9	0.102 ± 0.003	26.9 ± 0.3
	50	Before 1st pulse	0.38 ± 0.01	2.6 ± 0.1	21.6 ± 0.8	0.091 ± 0.003	21.0 ± 0.4
		Final	0.40 ± 0.00	5.4 ± 0.1	39.6 ± 1.9	0.156 ± 0.007	38.4 ± 0.5
Watermelon	15	Before 1st pulse	0.39 ± 0.03	6.6 ± 0.2 ±	17.3 ± 0.5	0.105 ± 0.003	14.8 ± 0.1
		Final	0.42 ± 0.01	14.7 ± 0.02	29.5 ± 0.1	0.123 ± 0.000	27.3 ± 1.3
	30	Before 1st pulse	0.36 ± 0.01	7.2 ± 0.2	22.4 ± 0.7	0.107 ± 0.003	21.4 ± 2.6
		Final	0.37 ± 0.01	11.7 ± 0.3	34.3 ± 0.9	0.142 ± 0.003	23.8 ± 2.9
	50	Before 1st pulse	0.26 ± 0.02	5.6 ± 0.2	23.8 ± 0.4	0.085 ± 0.003	22.3 ± 3.3
		Final	0.36 ± 0.01	9.7 ± 0.03	35.4 ± 0.1	0.151 ± 0.001	24.8 ± 1.9
Pepper	15	Before 1st pulse	0.70 ± 0.02	4.3 ± 0.1	8.6 ± 0.1	0.079 ± 0.001	7.3 ± 0.2
		Final	0.42 ± 0.01	16.8 ± 0.5	32.7 ± 1.1	0.114 ± 0.003	29.2 ± 1.3
	30	Before 1st pulse	0.43 ± 0.01	5.6 ± 0.2	18.4 ± 0.6	0.099 ± 0.003	14.8 ± 0.4
		Final	0.31 ± 0.02	13.1 ± 0.03	36.8 ± 2.3	0.119 ± 0.000	32.6 ± 1.6
	50	Before 1st pulse	0.31 ± 0.02	4.8 ± 0.1	20.8 ± 1.1	0.086 ± 0.002	15.9 ± 0.5
		Final	0.28 ± 0.03	11.3 ± 0.01	40.9 ± 0.4	0.129 ± 0.000	37.2 ± 0.2

**Fig. 2:**
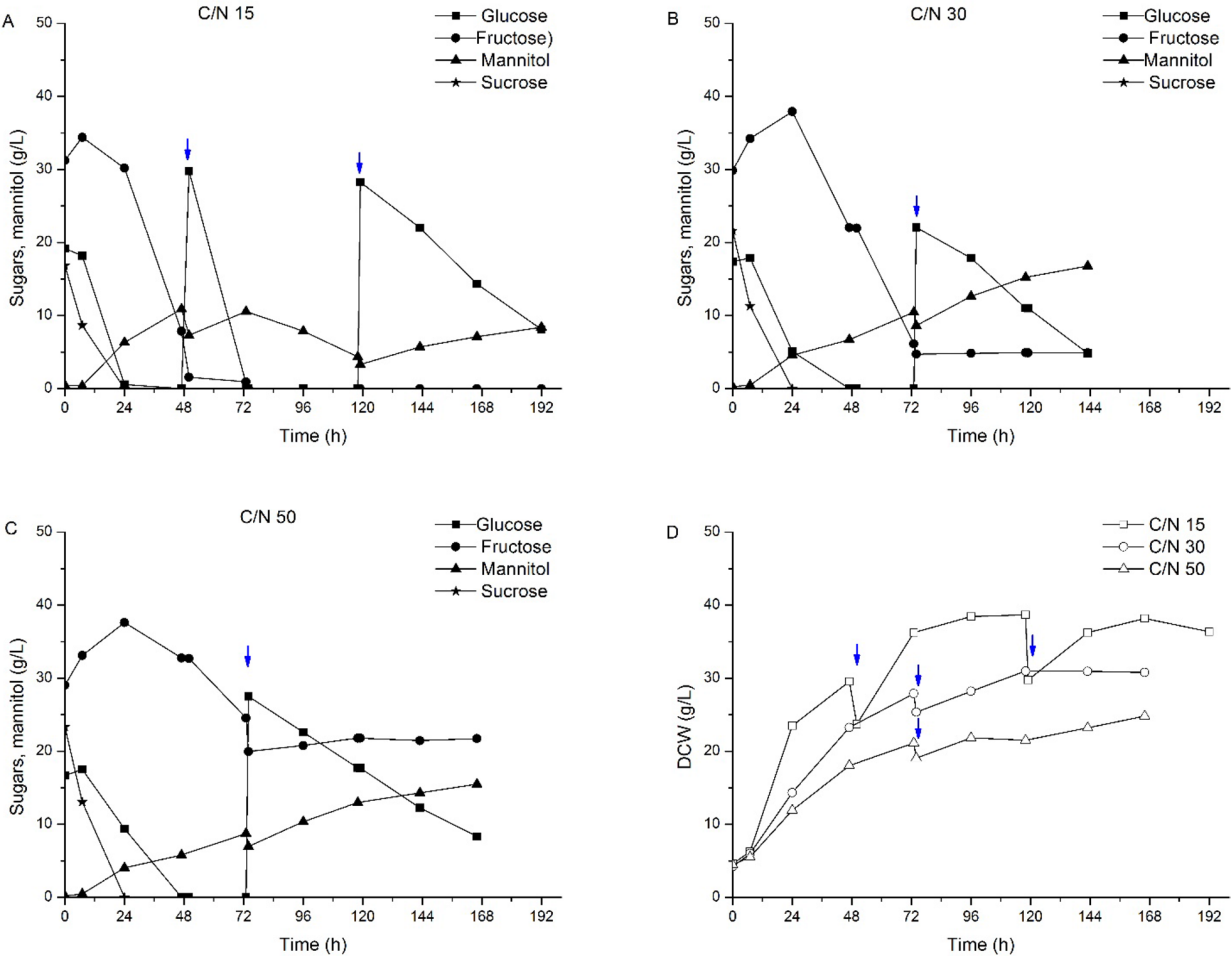
Time-course for sugars consumption and mannitol production from watermelon-derived media for different C/N ratios and cell biomass production ↓ Glucose pulse addition

**Fig. 3:**
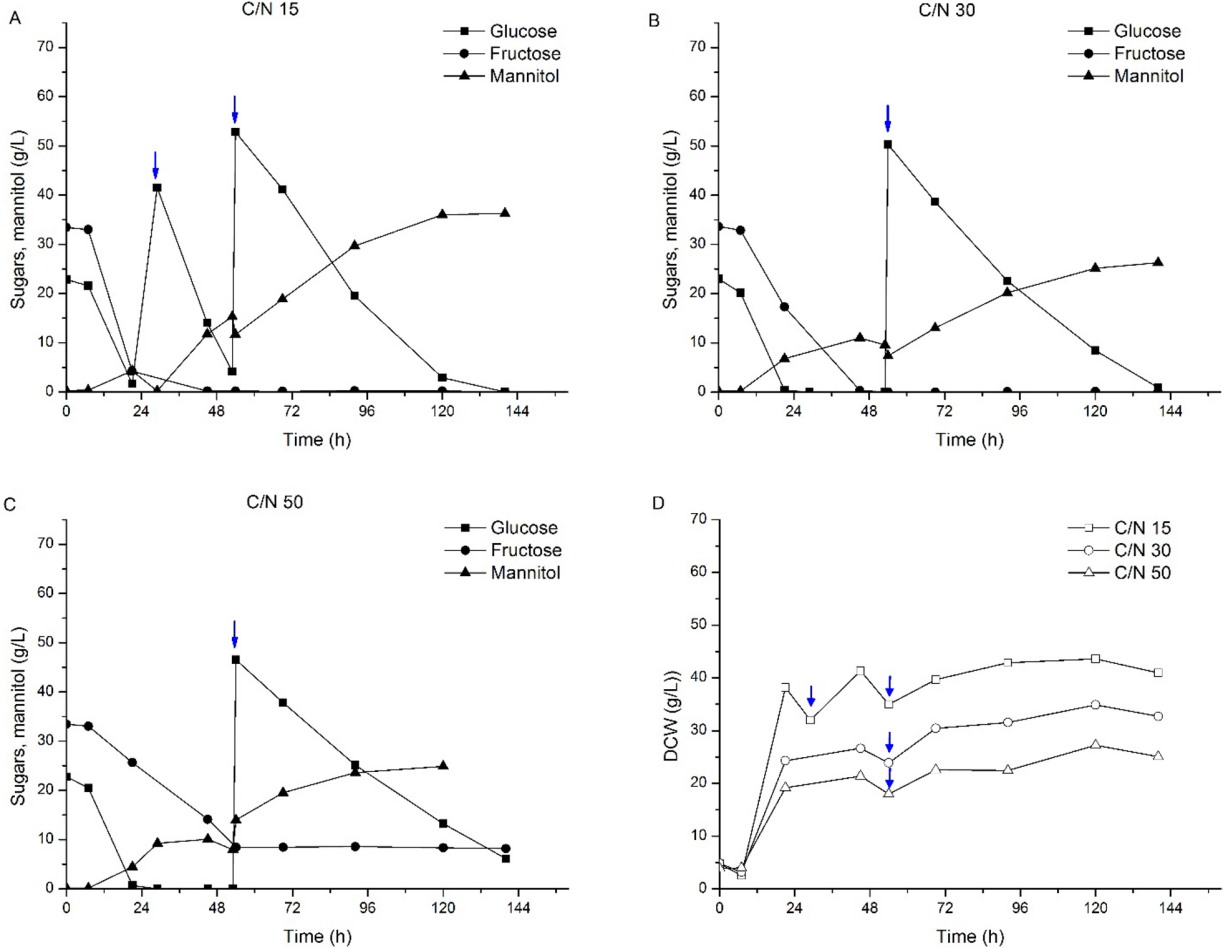
Time-course for sugars consumption and mannitol production from pepper-derived media for different C/N ratios and cell biomass production. **A**. ↓ Glucose pulse addition

A similar approach to that followed for discarded tomato media was applied to grow *C. curvatus* for lipid accumulation using the liquid fractions collected from watermelon and pepper. Sugar consumption and cell biomass production when culturing *C. curvatus* in media from discarded watermelon and discarded pepper are shown in Figs. 2, 3, respectively. Similar to discarded tomato, free glucose and fructose were identified as significant sugar components on pepper residue. In addition, discarded watermelon also showed a significant concentration of sucrose, about one-third of the total sugar content. This disaccharide was fully converted into the corresponding monomers within the first 24 h of the process (Fig. 2). This result is indicative of the presence of the invertase enzyme responsible for hydrolyzing sucrose into glucose and fructose. Since no external enzymes were added to the process, *C. curvatus CL6032* must be responsible for the production and secretion of such activity. Secretion of invertase activity by *C. curvatus* has been previously reported in literature [21], supporting sucrose hydrolysis in this study. The presence of sucrose when using the liquid fraction from discarded watermelon also revealed the preference of glucose over fructose by *C. curvatus* CL6032. As depicted in Fig. 2, sucrose hydrolysis resulted in the accumulation of free fructose; meanwhile, glucose concentration was constantly decreasing. This effect was more visible at higher *C*/*N* ratios due to the lower sugar consumption rates. The *C*/*N* ratio also influenced cell biomass concentration and intracellular lipid content. Total DCW production ranged between 21. And 29.6 g/L and 19.1–38.1 g/L for watermelon and pepper respectively, being the lower values representative of cells growing in media with higher C/N content as previously observed for discarded tomato-derived media (Figs. 2, 3).

On the other hand, the lipid content ranged between 17.3 and 23.8% and 8.6–20.8% when using watermelon and pepper residues, respectively (Table 2). In general, using the liquid fraction from discarded pepper as cultivation media for *C. curvatus* showed similar trends to discarded tomato media in terms of cell biomass production and intracellular lipid content. However, when using the liquid fraction from discarded watermelon, the intracellular lipid content was higher than discarded tomato or pepper. Lower concentrations of cell biomass and lipids (16.8 g/L DCW, 4.8 g/L of total lipids and 28.6% of intracellular lipid content) have been reported when using a watermelon peels waste media to grow the oleaginous microorganism *Syncephalastrum racemosum* [22]. On the other hand, an intracellular lipid accumulation of 39.6% with a lipid concentration of 2.9 g/L was reported by Hashem et al. [23] using the yeast *Lichthemia corymbifera* and a similar cultivation media (watermelon peels).

### Pulse-feeding cultivation

The use of agri-food waste as media for oleaginous yeast cultivation has also resulted in low lipid accumulation in previous works. For instance, the cultivation of *C. curvatus* NRRL Y-1511 in orange peel extract supplemented with ammonium sulphate and having a *C*/*N* ratio of 33 resulted in a total cell biomass concentration of 10.9 g/L and lipid content of 15% (*w*/*w*) (0.09 *g*/*g* of lipid yield) [15]. Therefore, developing alternative cultivation strategies is essential to increase the intracellular lipid content in oleaginous microorganisms when using agri-food residues. To reach this aim, the present work assessed a pulse-feeding strategy to raise the sugar content again before its exhaustion. This strategy was also designed to allow increasing the *C*/*N* ratio during the feeding phase while keeping a relatively high cell concentration. A highly concentrated pure glucose solution was used for the feeding phase and added right before sugar depletion (determined by low sugar levels or after observing an increase in the dissolved oxygen values). The pulse-feeding strategy was tested at different initial *C*/*N* ratio (15, 30 and 50) obtained from discarded tomato-derived media. The glucose solution was added once or twice, depending on the observed sugar consumption rates from the previous phase, so the fermentation could be extended up to 72–96 h. The lipid production capacity of *C. curvatus* under the pulse-feeding strategy was evaluated at the end of the process. This strategy increased the intracellular lipid concentration to 8.8 g/L, 4.7 g/L and 5.4 g/L at initial *C*/*N* 15, 30 and 50, respectively. From the total DCW, these lipid concentrations are equivalent to 29.4%, 29.2% and 39.6% in terms of total lipids and to 24.3%, 26.9% and 38.4% in terms of identified FAMEs content. The highest intracellular lipid concentration was observed in the media with the highest *C*/*N* ratio.

Oleaginous microorganisms can accumulate lipids up to 60–70% (*w*/*w*) DCW under favourable conditions, being the C/N ratio an important parameter when using sugar-based substrates [4]. Media supplementation with external sugars results in higher *C*/*N* ratios and can boost intracellular lipid accumulation in oleaginous yeasts. Fakas et al. [24] increased the intracellular lipid content in the yeast *Cunninghamella echinulata* from 9.7% to 42% of the total DCW after supplementing with glucose a tomato waste hydrolysate obtained with sulphuric acid treatment. This approach also improved the final lipid concentration by about four times, from 2.2 g/L to 8.5 g/L, reaching a sugar to lipid conversion yield of 0.11 g/g. In the present work, an intracellular lipid content close to 40% (*w*/*w*) DCW and a sugar to lipid conversion yield about 0.16 g lipid/g consumed sugars was observed by operating under a pulse-feeding strategy and using an initial *C*/*N* ratio of 50 (Table 2). This conversion yield is 40% higher to that reported by Fakas et al. [24], which might be explained by the higher initial *C*/*N* ratio that favors lipid accumulation. This statement is also supported by the fact that *C*/*N* 15 and *C*/*N* 30 media resulted in lipid conversion yields of 0.11 g lipid/g consumed sugar, even though *C. curvatus* cells showed a lower intracellular lipid content under these cultivation conditions (about 30% (*w*/*w*) from the total DCW).

Following the pulse-feeding approach, the intracellular lipid content in *C. curvatus* increased up to about 35% (*w*/*w*) and 41% (*w*/*w*) when growing on discarded watermelon and discarded pepper-derived media, respectively. The sugar to lipid conversion yields also increased up to 0.15 *g*/*g* and 0.13 *g*/*g*, respectively. Notwithstanding, despite increasing intracellular lipid content and conversion yields, lower total lipid concentrations were reached due to the lower overall cell biomass concentrations (Table 2). It is very interesting to note that *C. curvatus* produced mannitol in addition to lipids as a byproduct during the pulse-feeding process, independently of the residue used or the initial *C*/*N* ratio (Figs. 1, 2, 3). Mannitol started to accumulate in the media within the first stages of the process in the presence of sugars (either glucose, fructose or both).

In contrast, mannitol consumption could be also observed with low sugar levels. This result points out the ability of *C. curvatus* to produce this sugar alcohol and subsequently use it as a carbon source. The ability of *C. curvatus* to reduce fructose and/or oxidize mannitol was confirmed by measuring mannitol dehydrogenase activity in a crude cell extract during the mannitol production phase (cells were collected when mannitol concentration was about 10 g/L). The enzymatic activity measured for fructose reduction was 0.3 U/mg protein, while the enzymatic activity for mannitol oxidation was 1.1 U/mg protein. Mannitol production and consumption have been previously reported in other yeasts, such as *Yarrowia lipolytica* [25] and different species belonging to the *Wickerhamiella* and *Starmerella* genera [26]. The specific role of mannitol in yeast metabolism is mainly linked with redox balance and stress protection [25, 26]. Mannitol production can alleviate the redox imbalance of yeast cells by either recycling NADP^+^ when NADPH is in excess or providing the NADPH required during lipid production [25, 26]. In addition, mannitol production has also been related to stress-protective mechanisms to reactive oxygen species (ROS) [27] and/or to thermal stress [21]. In this work, depending on the cultivation media, maximum mannitol concentrations varied between 10 and 28 g/L. These significant concentrations also influence lipid production, thus, influencing sugar conversion yields. Nevertheless, mannitol has interesting applications in the food and pharmaceutical industries (e.g., excipient in pharmacy, anticaking and free-flow agent, lubricant, stabilizer and thickener, low-calorie sweetener) [28, 29] and, therefore, might also play an important role in these bioprocesses within a biorefinery context.

### Fatty acids profile

**Fig. 4:**
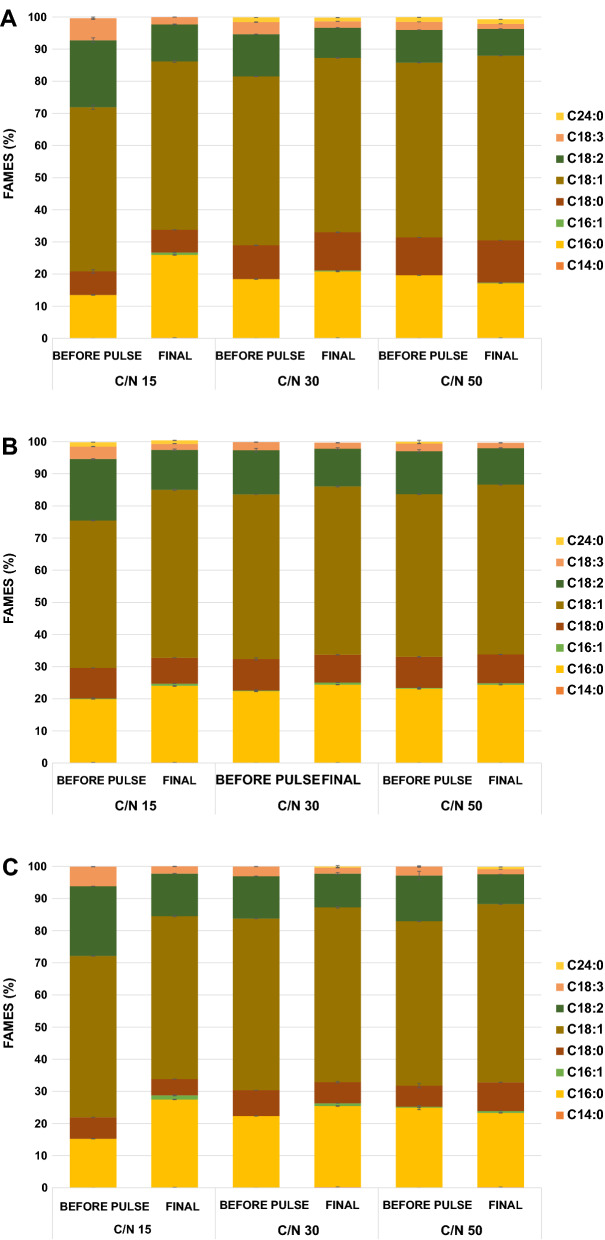
Fatty acid profile of *C. curvatus* grown on discarded vegetable-derived media at different culturing stages*.* **A** Tomato; (**B**) Watermelon; (**C**) Pepper

Another essential aspect of studying microbial lipid production to assess its technical application properly is the detailed FA profile. Figure 4 shows the FA profiles of cultivating *C. curvatus* CL6032 in different agri-food media before and after applying the pulse-feeding strategy. Overall, oleic and palmitic acids were identified as the dominant FAs before the pulse-feeding approach, accounting for 45.9–54.4% and 13.5–24.9% of the total FAMEs profile, respectively. After applying the pulse-feeding strategy, these ranges changed to 50.7–57.5% and 17.0–27.4%. Knowing the FA composition of the resulting microbial oil is essential to assess its potential applications, especially concerning biodiesel production. The presence of high levels of long-chain monounsaturated FA supports biodiesel quality as they are fluid at room temperature and could improve biodiesel flow characteristics.

Furthermore, a high percentage of polyunsaturated FAs is not suitable for biodiesel production because it not only affect the oxidative stability and quality of lipid-based biodiesel during storage but also lead to an increase in the viscosity of the final product [30]. Therefore, biodiesel should be ideally made from methyl esters of both saturated and monounsaturated FAs with a low content of polyunsaturated FAs. According to the FA profiles (Fig. 4), the resulting microbial oils were rich in monounsaturated FA (46.1–57.7%), while the concentration of saturated and polyunsaturated FAs represented up to 20.8–33.8% and 13.8–16.2%, respectively. Such FA profiles might therefore support using these microbial oils as an adequate feedstock for biodiesel production [31].

## Conclusion

Mechanical methods can easily extract sugars from discarded vegetable residues without needing previous and more expensive pretreatments. The liquid fractions resulting from fruits and vegetable residues represent an interesting carbon source for microbial oil production using oleaginous yeasts and for adequate cell growth or production of other compounds of interest, such as mannitol. The lipid accumulation in *C. curvatus* increases by using higher C/N ratios. Finally, the pepper-derived medium allowed the accumulation of higher lipid concentrations in *C. curvatus,* being the lipid profile from *C. curvatus* similar to that of vegetable oils commonly used for conventional biodiesel production. Optimizing the process, selecting a suitable cultivation strategy and using cost-effective carbon sources can lead to a more attractive process for biorefineries.

## Methods

### Raw material and oleaginous microorganism

Discarded vegetables from tomato, pepper and watermelon crops were used as substrate in this study. These residues were provided by Albaida Residues S.L. (Almería, Spain) and were obtained under greenhouse farming conditions. The carbohydrate content of the discarded vegetables was determined following methods described elsewhere [18]. The residues were crushed by homogenization in a Danamix TR/bM-330 industrial blender (SAMMIC; Azkoitia, Spain), and the resulting solid and liquid fractions were subsequently separated by centrifugation at 3000 rpm for 15 min, using a basket centrifuge RTL2BD (COMTEIFA; Barcelona, Spain). Liquid fractions were analyzed according to the methodology described below and stored at − 20 °C before their use.

The yeast *Cryptococcus curvatus* CL6032 obtained from the National Collection of Instituto de Salud Carlos III (BBN-ISCIII; Madrid, Spain) was used as an oleaginous microorganism. This strain was maintained in YPD agar plates with the following composition: 10 g/L yeast extract, 20 g/L peptone, 20 g/L glucose, and 20 g/L agar. Active cultures of this strain were obtained by inoculating a single colony into a 250 mL baffled Erlenmeyer flask containing 50 mL of YPD medium. Flasks were incubated in a rotary shaker at 180 rpm and 26 °C for 24 h. Cells were subsequently harvested by centrifugation at 3000* g* for 10 min and washed once with sterile water before inoculation.

### Microbial oil production

The soluble fractions obtained from discarded tomato, pepper and watermelon were used as the substrate for microbial oil production. In order to maximize intracellular lipid accumulation, different initial C/N ratios were evaluated. In this context, the soluble fractions were used directly (C/N 15) and mixed at 1:1 (C/N 30) or 1:2 (C/N 50) (liquid fraction: sugar solution) with a sugar solution containing fructose, sucrose and glucose at a known concentration to maintain the initial sugars concentrations measured in each residue-discarded media. Microbial oil production were assessed on 1-L Biostat B-Plus bioreactors (Sartorius; Göttingen, Germany), using an initial working volume of 400 mL of the corresponding filter-sterilized (Nalgene Rapid Flow^®^ filter 0.22 µm) liquid fractions. Assays were performed at 28 °C and pH 6 (pH was maintained with 2 M HCl and 2 M KOH), with a constant airflow of 1 vvm. Dissolved oxygen was constantly monitored and maintained above 20% (*v*/*v*) saturation level using variable stirring speed (500–1500 rpm). A 2% (*v*/*v*) antifoam solution (Antifoam 289, Sigma) was used as needed to prevent foam formation (2 mL added prior to inoculation). Cells were inoculated with an initial optical density (OD600_nm_) of 1–1.5 and cultivated following a pulse-feeding operational strategy for 140–168 h. Samples were withdrawn periodically to analyze sugars and cell biomass concentration.

Cultures were operated by a pulse-feeding strategy. In this context, sugar components were monitored throughout the process to determine the time point when reaching sugar depletion (i.e. when having low sugars concentration and/or when observing an increase in dissolved oxygen levels). After reaching sugar depletion, 50 mL of a concentrated, autoclave-sterilized glucose solution was added to level the sugar concentration up to the initial values (30–50 g/L depending on the substrate). Before glucose feeding, 50 mL of the corresponding fermented media were harvested to determine the intracellular lipid content and FA profile and evaluate conversion yields. Collected cells were harvested by centrifugation (5000 g, 4 °C, and 15 min), washed twice with sterile water, freeze-dried, and stored in a desiccator until further treatment.

### Analytical methods

Cell biomass concentration (DCW) was determined by filtering a known volume of cell suspension through a 0.22 µm nitrocellulose membrane (GE Healthcare; Germany). Cells were then washed with 4-times higher amount of water and dried in a microwave at 700 W for 10 min. DCW was determined as the difference between the weight of the empty dried filter and the dried filter with cells.

The concentration of sugars (*i.e.*, glucose, fructose, sucrose) and mannitol were determined by HPLC chromatography using an HPLC Waters Alliance 2695 system (Massachusetts, USA) equipped with a refractive index detector (detector 2414) and a Carbo Sep CHO 782 column (Transgenomic, Nebraska, USA), the latter operating at 70 °C with 0.5 mL/L ultrapure water.

Lipid content was determined by the gravimetric method according to Sha [32]. Briefly, 70 mg of freeze-dried cells were treated with 3.2 mL 4 M HCl at 55 °C for 2 h. Then 8 mL of a 2:1 (*v*/*v*) chloroform: Methanol solution was added, and the resulting solution was incubated at 20 °C for 3 h. After incubation, the solution was centrifuged (2000 g, 15 min) to recover the organic phase (chloroform). This step was repeated once by adding 4 mL chloroform to the aqueous phase, and all recovered organic phases were mixed up and subjected to evaporation in a TurboVap LV Evaporator under N_2_ to remove chloroform. The lipid content was expressed as g of lipid per 100 g of DCW and lipid yield was calculated as the ratio between total amount of lipid produced and amount of consumed sugars (g lipid/g consumed sugar).

The composition of fatty acid methyl esters (FAMEs) was analyzed following the method described by Van Wychen [33]. This method is based on transforming the FA present in lyophilized cells (~10 mg) into the corresponding FAMEs, which are collected by hexane and quantified by gas chromatography (GC). An Agilent GC 7890A (California, USA) equipped with a flame ionization detector (FID) and a split injector, and an Agilent polysiloxane capillary column DB-23 (length 30 m, 25 mm id, 1/20 split ratio). The injector and detector operated at 250 °C and 280 °C, respectively. Tridecanoic acid methyl ester was used as the internal standard, with a final 10 mg/mL concentration. FAMEs were identified by using a standard mixture [33].

Total nitrogen content of each corresponding fraction was determined according to the Kjeldahl method.

Mannitol dehydrogenase activity was determined in *C. curvatus* cells during the lipid accumulation phase. For that, cells were collected by centrifugation (3000 g, 5 min), washed twice with ice-cold 100 mM Tris-ClH buffer (pH 7.5) and disrupted in a FastPrep-24^™^ 5G instrument (MP NBiomedicals) using glass beads and 1 mL lysis buffer. Cells were subjected to 6 cycles of 30 s vortexing and 2 min ice. Then, lysed cells were centrifuged (16,000 g and 4 °C for 20 min) to collect supernatant. Mannitol dehydrogenase activity was measured in the collected supernatant at 25 °C. For that, both oxidation and reduction of NADH/NADPH and NAD^+^/NADP^+^ were determined in presence of fructose and mannitol, respectively. Oxidation of NADH or NADPH was monitored by measuring the absorbance at 340 nm, using 50 mM Tris–HCl (pH 8.5), NADH (1 mM) or NADPH (1 mM) and 25 µL protein extract (1.4 g/L of total protein). This reaction was triggered by adding fructose to a final concentration of 1 mM. On the other hand, reduction of NAD^+^ or NADP^+^ was monitored by measuring the absorbance at 340 nm, using 50 mM Tris–HCl buffer (pH 10), NAD^+^ or NADP^+^ (0.2 mM) and 25 µL of protein extract (1.4 g/L of total protein). In this case, the reaction was triggered by adding mannitol to a final concentration of 50 mM.

Total protein concentration was analyzed using the BCA Protein Assay Kit-Reducing Agent Compatible from Pierce^®^ (Thermofisher, Massachusetts, USA).

## Supplementary Information

**Additional file 1: Table S1.** Chemical composition of discarded vegetable residues in % dry weight basis.

## Data Availability

All data related to this study is reported within this article.

## Abbreviations

DCW: Dry cell weight
FA: Fatty acids
FAMES: Fatty acid methyl esters
ROS: Reactive oxygen species
SCO: Single cell oils
