# Supplementary material for: Efficient use of discarded vegetal residues as cost-effective feedstocks for microbial oil production
Source: Biotechnol Biofuels Bioprod. 2023 Feb 9;16:21. doi: 10.1186/s13068-023-02268-5 (PMC9912647; doi:10.1186/s13068-023-02268-5)
Supplement: Supplementary file 1 — Additional file 1: Table S1. Chemical composition of discarded vegetable residues in % dry weight basis. [file 13068_2023_2268_MOESM1_ESM.docx]

Additional file 1: Table S1. Chemical composition of discarded vegetable residues in % dry weight basis.

| **Component** | **Tomato** | **Watermelon** | **Pepper** |
| --- | --- | --- | --- |
| Total extract | 69.1 | 67.6 | 61.4 |
| Organic solvent-extract | 3.3±1.0 | 0.8 ± 0.58 | 3.6 ± 0.1 |
| Aqueous extract | 65.9±4.5 | 66.83 ± 4.3 | 57.8 ± 1.2 |
| Glucose | 17.6 ± 3.0 | 13.9+0.4 | 18.1 ± 0.5 |
| Fructose | 23.7 ± 2.0 | 29.1 ± 0.8 | 28.5 ± 0.0 |
| Sucrose | 0.3 ± 0.0 | 18.0 ± 1.0 | 2.2 ± 0.1 |
| Galactose | 0.3 ± 0.1 | 0.3 ± 0.04 | 0.6 ± 0.0 |
| Xylose | 0.3 ± 0.0 | 0.3 ± 0.01 | 0.4 ± 0.1 |
| Arabinose | 0.1 ± 0.1 | 0.3 ± 0.02 | 0.2 ± 0.0 |
| Mannose | 0.5 ± 0.1 | 0.2 ± 0.02 | 0.7 ± 0.0 |
| Cellulose (Glucan) | 8.0 ± 0.1 | 10.1±0.0 | 5.6 ± 0.12 |
| Hemicellulose^1^ | 4.6 ± 0.04 | 6.0 ±0.2 | 5.3 ± 0.1 |
| Acid-insoluble solid | 9.3 ± 0.8 | 5.2+0.9 | 17.1 ± 0.5 |
| Whole Ash | 6.9 ± 0.1 | 5.5+0.4 | 5.5 ± 0.1 |

^1^Xylan+Galactan+Arabinan+Mannan
